# Identification and expression profile of a putative basement membrane protein gene in the midgut of *Helicoverpa armigera*

**DOI:** 10.1186/1471-213X-7-76

**Published:** 2007-06-28

**Authors:** Jia-Lin Wang, Xiao-Juan Jiang, Qian Wang, Li-Jing Hou, Da-Wei Xu, Jin-Xing Wang, Xiao-Fan Zhao

**Affiliations:** 1 School of Life Sciences, Shandong University, Jinan 250100, China

## Abstract

**Background:**

The midgut undergoes histolysis and remodeling during the larval to adult transition in holometabolous insects, but the molecular mechanisms underlying this process are not well understood.

**Results:**

Using Suppression Subtractive Hybridization (SSH), we identified a 531 bp cDNA predicted to encode a 176 amino acid protein, which we call *hmg176*. Northern and western blot analysis suggested that high levels of *hmg176 *are expressed in the midgut during molting, but not during metamorphosis. HMG176 protein was detected by immunofluorescence within the membrane of fat bodies and the basement membrane of the midgut of both molting and feeding larvae, but not in metamorphically committed larvae. *In situ *hybridization revealed that *hmg176 *transcripts mainly localized to the columnar cells of the midgut. Interestingly, a non-steroidal ecdysone agonist, RH-2485, significantly upregulated expression of *hmg176*.

**Conclusion:**

These observations suggest that *hmg176 *encodes a larval-specific protein that may participate in sustaining larval midgut during larval development, possibly in response to ecdysteroid *in vivo*. This study will enlighten our understanding of the molecular mechanisms of tissue histolysis during metamorphosis.

## Background

The digestive tract of Lepidoptera is divided morphologically into three regions: the foregut, midgut and hindgut. The midgut, the largest portion of the digestive tract, is enclosed by a peritrophic membrane (PM), lined with midgut cells, basement membrane and muscular layers. Four kinds of gut cells are recognized in the Lepidopteran midgut, including columnar, goblet, regenerative, and endocrine cells. Columnar cells have many long, apical microvilli, which point toward the midgut lumen, and basal invaginations that comprise a basal labyrinth, which is involved in the secretion of digestive enzymes. Goblet cells have a large, goblet-shaped central cavity with a role in regulating the electrogenic K^+ ^secretion [1,2]. Regenerative cells located in the basal of the midgut regenerate new cells to replace the damaged cells and contribute pupal/adult midgut cells during metamorphosis. Endocrine cells, which are fewer in number, are usually basally located and extend up to the midgut lumen. Endocrine cells synthesize various polypeptide hormones that act during regenerative cell differentiation and to control secretion of specific digestive enzymes after feeding [3].

The PM is secreted by the lumenal surface of the midgut cells and serves to protect cells from mechanical injury or microbial infection. The PM is a semi-permeable structure composed of chitin and proteins [4]. There are two types of PM, defined on the basis of the site of synthesis. Type I PM delaminates from the entire midgut epithelium in response to feeding and the type of meal ingested. Type II PM is produced by a specialized region of the anterior midgut that is present throughout the life cycle [5]. During the larval molt, the midgut PM is shed and replaced by the gut cells.

During the larval to pupal metamorphosis, the midgut undergoes remodeling, during which programmed cell death (PCD) within the larval midgut and development of the pupal midgut epithelium occur simultaneously [6]. Insight into this process has come from analysis of other insect species. In *Drosophila*, PCD in the midgut is regulated by steroids [7,8], and the caspases Dronc and Drice are thought to regulate PCD in *A. aegypti *[9]. The morphological changes and PCD effectors during midgut remodeling have also been studied in *H. virescens *using histological and molecular techniques, and juvenile hormone analogs (JHA) inhibit both midgut remodeling and larval-pupal metamorphosis [10].

However, many aspects of midgut remodeling are still unclear. For example, the role of hemocytes and the regulation of their migration into the midgut are only beginning to be understood. Granular hemocytes have been shown to enter the midgut in metamorphically committed *Bombyx mori *larvae. Hemocytes are thought to accumulate in the midgut where they secrete type IV collagen, a major component of basement membrane, during larval to pupal metamorphosis [11]. In *Helicoverpa armigera *hemocytes have also been found to migrate into the midgut of 6th-72 h larvae and fat bodies at 6th-96 h larvae, tissues that will undergo histolysis at later larval stages [12]. The outer surface of the midgut, which faces the hemocoel, is surrounded by a basement membrane and muscular layers composed of bundles of inner circular and outer longitudinal muscles. The mechanisms by which the hemocytes enter these tissues are not known, although proteins expressed within the basement membrane might be predicted to play important roles.

Attempts to identify proteins differentially expressed in the midgut during larval molting by proteomic analysis have uncovered few proteins [13]. Suppression subtractive hybridization (SSH) is a powerful method for identifying differentially expressed genes. In this technique, cDNA from one population of cells/tissues is used as the "tester," to reveal cDNAs unique to a second population, the "driver" [14,15]. Using SSH with cDNA from molting 5th instar larvae with head capsule slippage, molting toward 6th instar (5th-HCS) as the tester and cDNA from feeding 6th instar larvae (6th-48 h post ecdysis) as the driver, we identified a cDNA encoding a 176 amino acid protein named *hmg176 *[16]. By BLAST search from GenBank [17], *hmg176 *shows 34% identity to *Drosophila *CG34026-PA, an uncharacterized gene. To characterize *hmg176*, we used Northern and western blot analyses, as well as immunohistochemistry and *in situ *hybridization to describe the expression profile of *hmg176 *during *Helicoverpa *development.

## Results

### Characterization of the hmg176 cDNA

**Figure 1:**
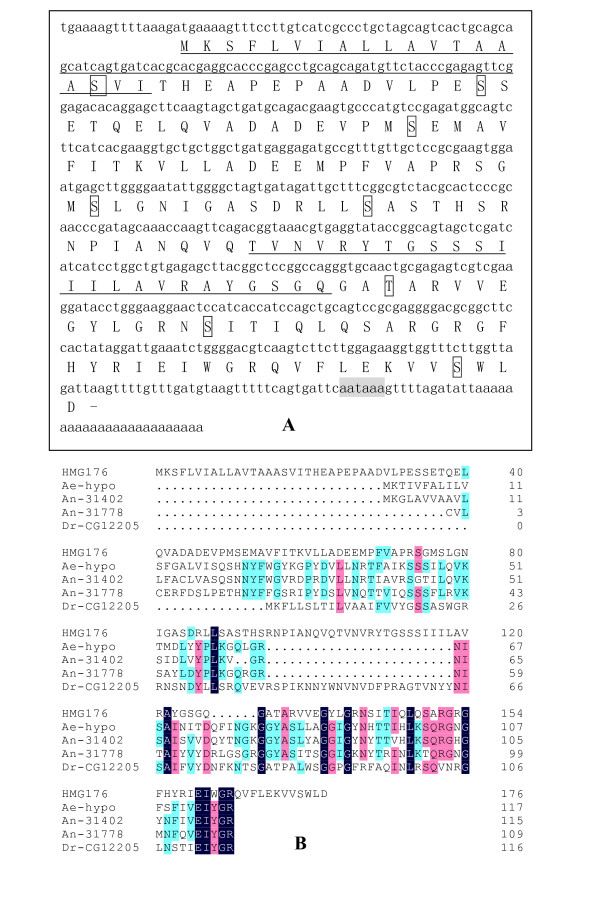
Bioinformatic analysis of *hmg176 *cDNA. **A**. Full-length *hmg176 *cDNA and predicted amino acid sequences. Underlined sequences correspond to predicted transmembrane helices. A possible signal peptide cleavage site is located between amino acids 16 and 17. Boxed amino acids are putative phosphorylation sites. The shadowed sequence is a poly A tail signal sequence. **B**. Alignment of HMG176-like proteins from other species. ENSANGP00000031402 is from *A. gambiae *32% identity, CG34026-PA is from *D. melanogaster *34%, ENSANGP00000031778 is from *A. gambiae *39%, GA12205-PA is from *D. pseudoobscura *31%, conserved hypothetical protein from *Aedes aegypti *31%. (E-Value < 0.001).

A full-length *hmg176 *cDNA was obtained by Suppression Subtractive Hybridization (SSH) using the molting 5th larval midgut (5th-HCS) as the tester and the feeding 6th larval midgut (6th-48 h) as the driver. The cDNA included a 531 bp open reading frame (ORF), a 75 bp sequence upstream of the ORF and a 77 bp sequence downstream, followed by a poly A tail. The cDNA encodes a 176 amino acid protein with a theoretical pI of 5.4 and a predicted molecular weight of 19 kDa. Amino acids (aa) 1–19 are predicted to comprise a signal sequence, and includes a putative cleavage site between aa 16 and 17. In addition, a putative transmembrane region is located between aa 104 and 127. The presence of poly A tail addition sequence strongly suggests that a full-length cDNA was cloned. Seven predicted serine phosphorylation sites and one threonine phosphorylation site were identified, but no N- or O-linked glycosylation sites were present (Fig. 1A). By BLASTX analysis, *hmg176 *is similar to other genes, with E-value < 0.001, including ENSANGP00000031402 from *Anopheles gambiae *(32%), CG34026-PA from *Drosophila melanogaster *(34%), ENSANGP00000031778 from *A. gambiae *(39%), GA12205-PA from *D. Pseudoobscura *(31%), and a conserved hypothetical protein from *Aedes aegypti *(31%) (Fig. 1B). Analysis by ProtFun 2.2 predictions [18] suggests that HMG176 is probably a cell-envelope protein.

### Expression profile of hmg176

**Figure 2:**
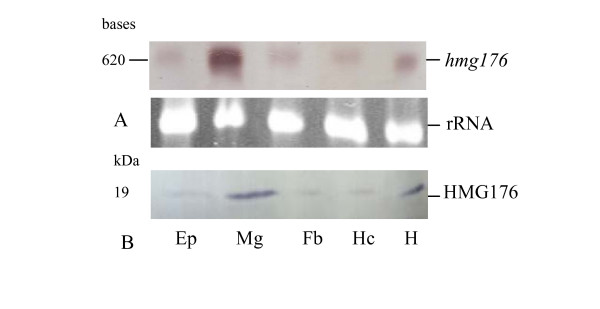
Expression analysis of *hmg176 *in molting 5th instar larvae with head capsule slippage (HCS). A) Northern blot analysis of *hmg176 *in various tissues. B) Western blot analysis of HMG176 protein in various tissues. Ep, epidermis; Mg, midguts; Fb, fat bodies; Hc, hemocytes; H, head.

To verify the tissue specificity of *hmg176 *expression suggested by the SSH, total RNA was isolated from the epidermises, midguts, fat bodies, hemocytes and heads (including fat body, brain, foregut, muscle, epidermis and various glands around this portion) of molting 5th instar larvae with head capsule slippage (5th-HCS, molting toward 6th instar) and analyzed by Northern blot. The *hmg176 *with 620 bases was primarily detected in the midgut, and lower level transcripts were detected in all other tissues, consistent with the SSH study (Fig. 2A). Western blot analysis revealed a similar distribution of HMG176 protein in all tested tissues (Fig. 2B).

**Figure 3:**
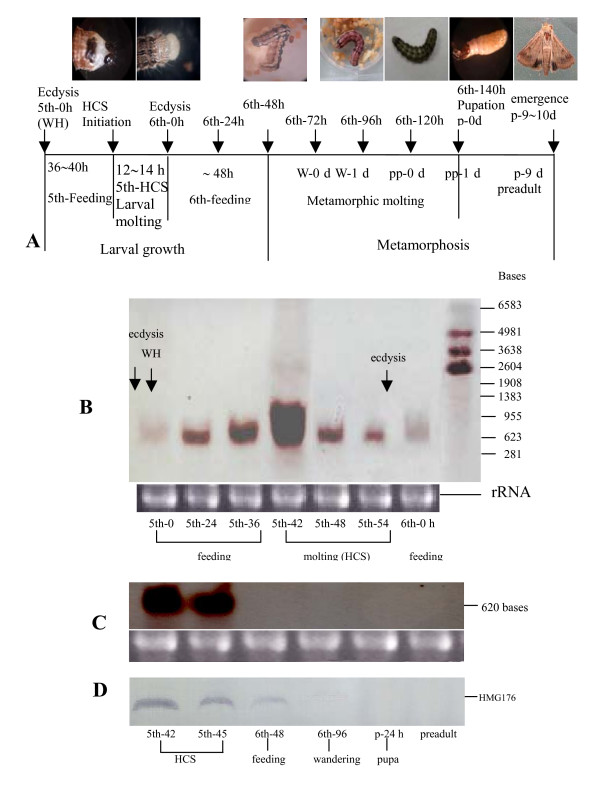
Expression profile of *hmg176 *in the midgut during larval development. A) Overview of developmental stages of *H. armigera*. WH, white head capsule; HCS, head capsule slippage. B) Northern blot analysis of *hmg176 *expression in the larval midgut during larval molting. 5th-0, 5th-24, 5th-36, 5th-42, 5th-48, 5th-54, and 6th-0 h are the development stages of each instar larvae; C) Northern blot analysis of *hmg176 *expression in the midgut during metamorphosis. 5th-42 (HCS), 5th-45 (HCS), 6th-48 (feeding), 6th-96 (W-1 d), p-24, and preadult are the development time points of midgut from molting 5th instar larvae (HCS) to preadult. D) Western blot analysis of HMG176 expression in the midgut during metamorphosis. (Same stages as in panel C).

The expression profile of *hmg176 *throughout development was further analyzed in midguts from 5th instar larvae to preadult by Northern blot (Fig. 3A). Northern blot analysis showed that expression of *hmg176 *was much lower in 5th-0 h larvae (0 h after ecdysis), which have recently removed the old cuticle and have a white head capsule (WH). *hmg176 *expression increased slowly with feeding, and peaked at the beginning of HCS stage in 5th-42 h larvae, the stage at which larvae initiate head capsule slippage (5th-HCS). Subsequently, *hmg176 *transcription decreased to lowest levels by ecdysis of the 6th instar (Fig. 3B). These results are consistent with the proposal that the expression of *hmg176 *is upregulated specifically during larval molting although it increases with feeding in larvae.

In contrast to the high levels expressed during the larval molting stage, *hmg176 *was not detectable from 6th-24 h larvae (24 h after ecdysis, data not shown), 6th-48 h larvae (48 h after ecdysis), 6th-96 h larvae (wandering), p-24 h pupae (24 h after pupation), or in preadults (P-9 d after pupation, one day before adult ecdysis) when larvae are metamorphically committed, until late pupal stage (preadult) (Fig. 3C). These results suggest that *hmg176 *is specifically expressed during larval stages, but not during metamorphosis.

To verify the expression pattern of this gene during metamorphosis, we performed a Western blot analysis of midguts from molting 5th larvae (5th-HCS), feeding 6th larvae (6th-48 h), wandering 6th-96 h larvae (6th-96 h), earlier pupae (p-24 h), and later pupae (preadults, P-9 d). Consistent with our RNA analyses, HMG176 protein was detected in the molting 5th (5th-HCS) larval midgut and in the feeding 6th-48 h larval midgut. However, the amount of protein detected in the feeding 6th-48 h larval midgut was much lower than in molting 5th larvae. In contrast, no obvious protein was detected in the midguts of wandering 6th-96 h larvae, pupae (p-24 h), or and preadults (Fig. 3D). These observations strongly suggest that HMG176 plays a specific role during larval growth and development.

### HMG176 expression pattern in the midgut

**Figure 4:**
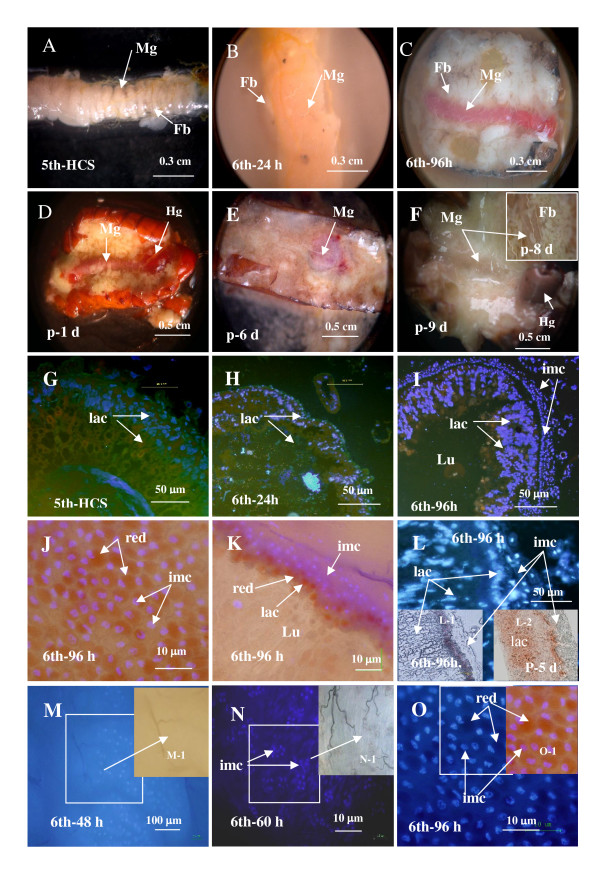
Morphological changes in midgut morphology during development. Panels A-F are from larvae of 5th-HCS, 6th-24 h (feeding), 6th-96 h (wandering), p-1 d (pupa 24 h after ecdysis), p-6 d (pupa 6 d) and preadult (p-9 d). Mg, midgut; Fb, fat body; Hg, Hindgut. Panels G-I, cryosections of midguts from molting 5th instar (5th-HCS), feeding 6th instar (6th-24 h) and 6th-96 h larvae stained with DAPI. Panels J-L, location of red spots. J) Midgut from 6th-96 h larvae (surface, UV and white light). K) Red larval midgut and the imaginal cells of the 6th-96 h midgut (dissected, UV and white light). L) Cryosection of 6th-96 h midgut observed under UV light. L-1 and L-2) Midguts from 6th-96 h larva and p-5 d pupa and observed directly under white light without other treatment. Panels M-O) DAPI in whole mount midguts from 6th-48 h, 6th-60 h, and 6th-96 h larvae, showing formation of the imaginal cell layers. M-1 and N-1) white light images. O-1) Merged white and UV light images. lac, larval midgut; imc, imaginal cells; Lu, lumen of midgut; red spot.

To determine the distribution of HMG176 in the midgut, we began by observing morphological changes in the midgut during development. We observed dramatic changes in midgut morphology during larval development and metamorphosis. The midgut of molting 5th larvae (5th-HCS) appeared helix-like shape and was covered with a transparent membrane. The midgut of feeding 6th larvae (6th-24 h after ecdysis) was full of food. Interestingly, the 6th-72 h midgut of wandering larvae (72 h after ecdysis, W-0 d) became red, as was the entire body. The red color of the midgut deepened in 6th-96 h wandering larvae (W-1 d), and this persisted in 6th-120 h-140 h prepupae when larvae had contracted their legs and ceased crawling (W-2-3 d), until earlier pupal stage (p-1-2 d). Along with the histolysis of the larval midgut and adult midgut remodeling, the midgut assumed the morphology of a large round vesicle, containing a red substance from the decomposed larval midgut at p-3-p-8 d, which slowly accumulated in the hindgut (Fig. 4A–F).

Through further analysis of the microstructure of the midgut, we noted that the midgut of wandering 6th-96 h larvae was quite different from both molting 5th and feeding 6th larval midguts. One difference was that there were several extra layers of cells surrounding the outside of the midgut in wandering 6th-96 h larvae that are not present in the molting 5th or feeding 6th larval midgut (Fig. 4G–I). The second difference was the red color in the wandering larval midgut. Microscopy showed that the red color was due to the presence of red spots (Fig. 4J). The red spots were located in the larval midgut underneath the imaginal cells, but did not overlap with imaginal cells nuclei (Fig. 4K). Analysis of cryosections, prepared from larvae throughout development, showed that the red color was first detectable in the basal cells of the larval midgut of wandering larvae and eventually seemed to expand to the entire larval midgut around the time of histolysis during metamorphosis (Fig. 4L-1, L-2). Groups of imaginal cells appeared before the midgut turned red at about 6th-60 h (Fig. 4N), and began to form cell layers around the time that the midgut became red at 6th-96 h (Fig. 4O). The nuclei of the imaginal cells, distributed on the surface of the midgut, were examined using DAPI staining.

**Figure 5:**
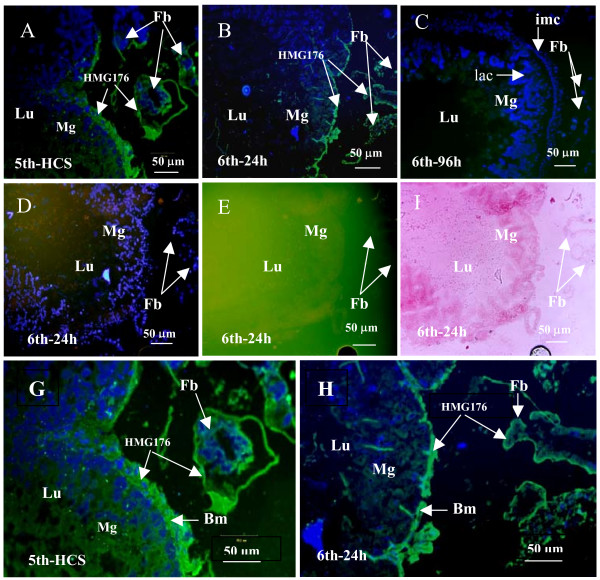
Immunocytochemical localization of HMG176 expression in the midgut. Panels A-C are midguts from molting 5th instar larva (5th-HCS), feeding 6th instar larva (6th-24 h), and 6th-96 h (wandering) larva. Panels D-F are negative control without anti-HMG176 sera, midgut from feeding 6th instar larva (6th-24 h). Panels G and H are the magnified A and B. Lu, lumen of midgut; Mg, midgut; Fb, fat body; Hg, Hindgut; imc, imaginal cells; lac, larval midgut cell. Bm, basement membrane.

Expression and localization of HMG176 correlated with the morphological changes in the midgut. Immunohistochemical analysis revealed a strong HMG176 signal in the basement membrane of the 5th-molting larval midgut (5th-HCS), suggesting that HMG176 contributes to the basement membrane surrounding the midgut. In addition, a strong signal was detected in the membrane of the fat bodies of molting 5th larvae. Relatively lower levels of HMG176 protein were detected in the midgut and fat bodies of feeding 6th larvae (6th-24 h), while no signal was detected in midgut or fat bodies of wandering 6th-96 h larvae (Fig. 5A–F). Similarly, no signal was detected in the midgut and fat body of p-6 d pupa and p-9 d preadult (data not shown). These results are consistent with our previous analyses.

**Figure 6:**
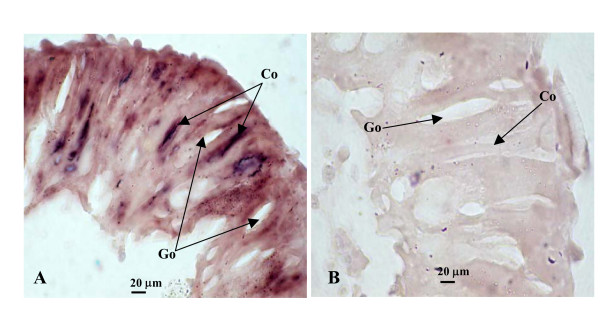
*hmg176 in situ *hybridization in the midgut. A) Midgut from molting 5th instar larva (HCS), antisense RNA probe. B) Same stage as in panel A, sense RNA probe as negative control. Co, clumnar cells; Go, goblet cells.

We also examined the expression of *hmg176 *in molting 5th larval midgut cells by *in situ *hybridization using digoxigenin-labeled RNA antisense and sense probes to *hmg176*. We detected a strong positive signal in the columnar cells of the midgut (Fig. 6A). In contrast, no signal was detected in negative control samples with hybridized with sense probes. Thus, *hmg176 *is expressed predominantly in columnar cells of the midgut (Fig. 6B).

### Effects of RH-2485 on the expression of hmg176

**Figure 7:**
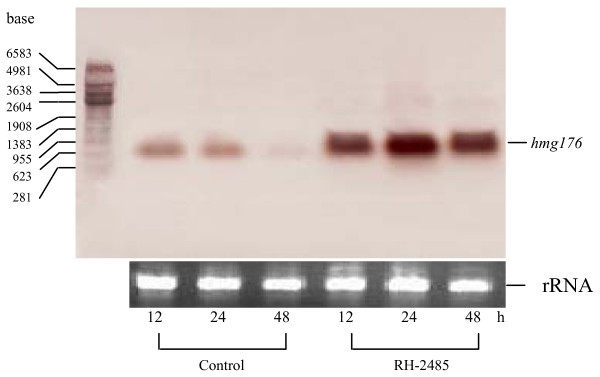
Northern blot analysis of RH-2485-mediated expression of *hmg176 *in the larval midgut. Control, midguts from 6th instar larvae (within 1 h post-ecdysis with white head capsule), injected with carrier alone 12, 24 and 48 h after injection. RH-2485, midguts from same stages injected with 1 μg RH-2485.

Since *hmg176 *expression was upregulated during molting, we hypothesized that it is regulated by 20-hydroxyecdysone (20E), an important molting hormone. To test this hypothesis, the 20E agonist RH-2485 was injected into the hemocoel of freshly ecdysed 6th instar larvae (with white head capsule) to examine its effect on the expression of *hmg176*. Figure 7 shows that *hmg176 *was upregulated 12 h post-injection, with maximal expression at 24 h post injection that declined thereafter (Fig. 7).

## Discussion

Here we showed the expression profile of *hmg176 *during *Helicoverpa *larval development. Our results demonstrate that *hmg176 *expression in the midgut increased towards HCS and peaked at the beginning of HCS stage in 5th-42 h larvae (5th-HCS), which suggests that *hmg176 *is upregulated specifically during larval molting. *hmg176 *expression appeared to cease beginning around 6th instar, and remained undetectable throughout preadult stages, suggesting that *hmg176 *is specifically expressed during larval stages, but not during metamorphosis or after the last larval ecdysis.

Our data further suggest that this protein might be involved in sustaining larval midgut during larval feeding and protecting the midgut from histolysis and remodeling. We detected expression of *hmg176 *in both the basement membrane of the midgut and the outer membrane of the fat body where it might function. This proposal is corroborated by our computational analysis, which suggested that *hmg176 *is a cell-envelope protein. However, the precise role of this and other *hmg176*-like proteins has not been reported.

In *Manduca*, HCS occurs when levels of ecdysteroid peak, and larval ecdysis occurs at when levels of juvenile hormone (JH) peak and when 20E begins to decline [19]. Some molting regulating genes are known to be expressed before HCS. For example, levels of the transcription factor MHR3 are thought to rapidly increase about 6 h before HCS and to peak 2–5 h before HCS [20]. 20E levels of *Helicoverpa *reaches peak levels during 5th and 6th larval molts and decreases to lower level during ecdysis [21], which is similar to the expression pattern of *M. sexta *[19,22]. We observed that *Helicoverpa *head capsule slippage (HCS) initiated at the 5th 36–40 h stage and is maintained for 12–14 h, which coincides with the peak of 20E levels. The fact that *hmg176 *expression obviously increased and peaked at HCS, and declined immediate after ecdysis, suggests that this gene is regulated by 20E during larval molting.

RH-2485 is a member of a family of non-steroidal ecdysone agonists, and mimics the action of 20E. The molecular mechanisms that cause premature, incomplete molting have been elucidated [23,24]. We previously reported that RH-2485 cause premature molting of *Helicoverpa *larvae by inducing expression of HHR3 [25,26]. We observed that *hmg176 *was upregulated by RH-2485, suggesting that *hmg176 *may normally be regulated by 20E *in vivo*.

The details of pupal/adult midgut formation are not completely known. In *H. virescens*, the pupal/adult midgut mainly consists of imaginal cells supported by layers of muscles and tracheal cells. The imaginal cells appeared at 96 h after ecdysis to the final larval instar and they formed a layer at 180 h outside of the larval midgut where they proliferate to form the pupal/adult midgut [10]. Recently, Ohlstein and Spradling [27] reported that the adult *Drosophila *posterior midgut is maintained by pluripotent stem cells. The midgut cells are continuously replenished by a distinct population of intestinal stem cells. Notch signaling is required for the differentiation of intestinal stem cells daughter cells. The identification of *Drosophila *intestinal stem cells demonstrates a possible mechanism for the maintenance of the adult midgut. We observed the groups of imaginal cells appeared and formed cell layers of the pupal/adult midgut when *hmg176 *stopped expression, which indicates the cessation of *hmg176 *is correlated to the formation of the pupal/adult midgut.

Hemocytes have been found to enter the metamorphically committed larval midgut to participate the tissue remodeling [11]. In *Bombyx*, granular hemocytes accumulate in a layer surrounding the midgut epithelium during pupal metamorphosis. It has also been reported that the number of granular hemocytes increases in circulating hemolymph and hemocytes migrate from hematopoietic organs to the tissues that will undergo histolysis during larva development [28]. Interestingly, regions of hemocyte accumulation are correlated with increased expression of the cathepsin B-like proteinase, which might play a key role in fat body and midgut histolysis during metamorphosis in *H. armigera*. Cathepsin B has been shown to be involved in the break down of adult fat bodies in *H. armigera *[29,12]. Because *hmg176 *expression ceases when hemocytes enter the midgut during metamorphosis, this protein might be expected to help maintain the midgut by protecting it from invading hemocytes. The disappearance of *hmg176 *expression at the same time as the basement membrane might render the midgut more permeable to hemocytes, thus providing a mechanism for invasion of the midgut and fat body during metamorphosis.

## Conclusion

We have presented the expression profile of a putative basement membrane protein gene. The expression of *hmg176 *is upregulated by 20E *in vivo *and appeared to cease beginning around 6th instar, and remained undetectable throughout preadult stages, suggesting that *hmg176 *is specifically expressed during larval stages, but not during metamorphosis or after the last larval ecdysis. We propose that this protein plays a role in maintenance and protection of larval tissues during certain stages, while subsequent downregulation of this gene would then allow the histolysis and remodeling of the midgut and fat body.

## Methods

### Insects

The cotton bollworm, *Helicoverpa armigera*, was reared in our laboratory on an artificial diet in a 14 h light/10 h dark regime [30].

### RNA isolation

Total RNA was isolated from various tissues at different developmental stages using Unizol reagent according to the manufacturer's protocol (Biostar, Shanghai, China). mRNA was isolated using Quickprep™ Micro mRNA Purification Kit (Amersham, Buckinghamshire, England).

### Suppression subtractive hybridization (SSH)

Suppression Subtractive Hybridization was performed using the Clontech PCR-Select cDNA Subtraction Kit (BD Bioscience Clontech). Midguts from molting 5th instar larvae (5th-HCS) with head capsule slippage were used as testers and those from feeding 6th instar larvae (6th-48 h post ecdysis) were used as drivers. Total RNA was isolated from midguts and cDNA was synthesized according to the method (Super SMART™ PCR cDNA Synthesis Kit, Clontech, Mountain View, USA). cDNA was then processed by restriction digestion of *RSA*I, adaptors were added, hybridized in two rounds, followed by two rounds of PCR amplification at the condition of 94°C 30 S, 66°C 30 s, 72°C 1.5 min, 27 cycles. PCR products were ligated into pGEM-T Easy vector (Promega, Madison, USA) followed by random sequencing of the clones.

### Prediction and characterization of the full-length cDNA

The HMG176 was analyzed by software in ExPASy Proteomics Server [31], including Compute pI/Mw, signal peptide with SignalP, TMpred, NetPhos, NetNGlyc, and NetOGlyc. Protein similarity searches were performed with Basic Local Alignment Search Tool (BLASTP) [32].

### Northern blot analysis

Northern blot analysis was carried out according to the protocol supplied by Roche (Boehringer Mannheim, Mannheim, Germany). Antisense Digoxigenin-labeled RNA probe was prepared using full length HMG176 cDNA inserted into pGEM-T Easy as a template. Approximately 10 μg total RNA was isolated from various tissues and electrophoresed on an agarose gel. After transfer to Nylon membrane by capillary transfer, blots were UV crosslinked for 10 min, prehybridized for 1 h at 68°C, hybridized with digoxigenin labeled antisense RNA probe (100 ng/ml) for 12 h at 68°C, washed twice for 5 min with 2× wash solution (2 × SSC, 0.1% SDS) at room temperature, and twice for 15 min with 0.1× wash solution (0.1 × SSC, 0.1% SDS) at 68°C, blocked for 30 min at room temperature, and detected with nitroblue tetrazolium chloride (NBT)/5-Bromo-4-chloro-3-indolyl phosphate (BCIP) according to the manufacturer's protocol.

### Preparation of antiserum against HMG176

The HMG176 was expressed in *Eschrechia coli *BL21 using pGEX-4T-1 vector (Amersham Buckinghamshire, England). Rabbit polyclonal antiserum against HMG176 was prepared using recombinant protein purified from *E. coli *by SDS-PAGE. Then the protein band was gel-excised and dialyzed into running buffer electrically. One hundred μg protein was homogenized with 1 ml of complete Freund's adjuvant. This was injected hypodermically into the back of the rabbit, once a week for three weeks. Three booster injections were given by ear blood vessel once a week for another three to five weeks. The specificity of the antiserum was examined by western blot and the antiserum was used in all immunoassay experiments.

### Immunohistochemistry

Midguts were excised and embedded in Frozen Section Medium (Kalamazoo, United States). After freezing in liquid nitrogen, 7 μm cryosections were cut with a MICROM HM550 cryostat microtome and placed on glass slides. The sections were then dried at room temperature overnight, fixed with cold acetone (-20°C) for 10 minutes, and washed 3 times with 1 × PBS (140 mM NaCl, 10 mM sodium phosphate, pH 7.4). Sections were then incubated with a primary antibody against HMG176 diluted to 1:100, and then goat anti-rabbit-ALEXA 488 (Eugene, United States) diluted to 1:1000 in 1 × PBS with 2% bovine serum albumin at room temperature for 2 h. Nuclei were stained with 4'-6-Diamidino-2-phenylindole dihydrochloride (DAPI, 1 μg/ml in water, San Jose, United States) for 10 min. Negative controls were treated the same, but pre-immune rabbit serum used in place of the antiserum against HMG176. Fluorescence was detected with an Olympus BX51 fluorescence microscope.

### *In situ *hybridization

In situ hybridization was performed according to the protocol supplied by Roche. Briefly, midguts were excised from molting 5th instar larvae, fixed with 4% paraformaldehyde overnight, embedded in paraffin, and sectioned as described above. After dehydration by ethanol and xylene, tissue sections were prehybridized for 10 min at 37°C, then hybridized with Dig-labeled antisense or sense RNA probes (400 ng/ml) at  42°C overnight. Sections were then washed sequentially (2 × SSC, 1 × SSC, and 0.1 × SSC) twice for 15 min each, then incubated with Anti-Dig phosphatase AB, and then NBT/BCIP for color development.

### Effect of RH-2485 on the expression of hmg176

RH-2485 (Rohm and Haas Company, Spring House, Pennsylvania, USA) was dissolved in isopropanol at 10 mg/ml. 6th instar larvae 1 h post-ecdysis (after shedding of old cuticle and emergence of new white head capsule) were injected with 1 μg RH-2485 in 5–10 μl PBS. Total RNA was isolated from larval midguts at 12, 24 and 48 h after injection for Northern hybridization analysis. As a negative control, an equal amount of isopropanol in PBS without RH-2485 was injected.

## Authors' contributions

Jia-Lin Wang carried out immunoblotting. Xiao-Juan Jiang performed Northern blot. Qian Wang completed immunohistochemistry. Li-Jing Hou did induction of RH-2485. Da-Wei Xu prepared polyclonal antibody. Jin-Xing Wang participated in the design and coordination of the work. Xiao-Fan Zhao participated in the design of the study and drafted the manuscript. All authors read and approved the final manuscript.

## Accession numbers

The nucleotide sequence reported in this paper has been submitted to GenBank with accession number [GenBank: DQ847154].
